# Long-lasting effects of the COVID-19 pandemic on lifestyle and body weight: results of representative cross-sectional surveys in adults in Germany

**DOI:** 10.1186/s12889-024-18680-x

**Published:** 2024-04-30

**Authors:** Hans Hauner, Carmen P. S. Blanken, Christina Holzapfel

**Affiliations:** 1 Institute of Nutritional Medicine, School of Medicine and Health, Technical University of Munich, Georg-Brauchle-Ring 62, Munich, 80992 Germany; 2 Else Kröner Fresenius Center for Nutritional Medicine, Technical University of Munich, Georg-Brauchle-Ring 62, Munich, 80992 Germany; 3 Department of Nutritional, Food and Consumer Sciences, Fulda University of Applied Sciences, Fulda, Germany

**Keywords:** Obesity, Lockdown, Nutrition, Physical activity, Mental health

## Abstract

**Background:**

The COVID-19 pandemic severely affected people’s daily lives and health. Few studies have looked into the persistence of these changes. In the current study, we investigated to what extent changes in lifestyle and body weight were sustained after two years of restrictions.

**Methods:**

We performed two representative online surveys among adults living in Germany. The first survey (S1) was performed in April 2021; the second survey (S2) in June 2022. The questionnaire focused on changes in physical activity, dietary habits, body weight, and mental stress levels. The data were weighted to optimally represent the general population of Germany. Using Chi-square tests, results were compared between the two surveys, and – per survey – between subgroups based on sociodemographic factors and mental stress levels. Furthermore, binomial logistic regression was performed to identify factors associated with weight gain.

**Results:**

A total of 1,001 (S1) and 1,005 (S2) adults completed the survey, of which 50.4% were men and 49.6% were women in both surveys. Mean body mass index (BMI) at the time of the survey was 27.4 ± 6.0 kg/m^2^ (S1) and 27.1 ± 5.5 kg/m^2^ (S2). Reduced physical activity was reported by 52% of the participants in S1 and by 40% in S2 (*p* < .001). Moderate to severe stress was reported by 71% of the participants in S1 and by 62% in S2 (*p* < .001). Less healthy eating compared to before the pandemic was reported by 16% of the participants in S1 and by 12% in S2 (*p* = 0.033). Weight gain was reported by 40% of the participants in S1 and by 35% in S2 (*p* = 0.059). Weight gain was associated with higher BMI, reduced physical activity levels, less healthy nutrition and increased consumption of energy-dense food.

**Conclusions:**

Our results indicate that two years and three months after the start of the COVID-19 pandemic, the adverse effects on health-related lifestyle factors and body weight still existed, albeit to a lesser degree than directly after the first year of the pandemic. Targeted strategies are needed to better support the population subgroups most likely to change their lifestyle in unfavorable ways when faced with disruptions of their everyday lives.

**Supplementary Information:**

The online version contains supplementary material available at 10.1186/s12889-024-18680-x.

## Background

The COVID-19 pandemic posed an extraordinary threat to public health when it started to spread out in early 2020. In most European countries, a first lockdown was set into place in March 2020, and over the consecutive two years – depending on regional infection rates and policies – more lockdowns and distinct restrictions followed.

Numerous restrictive measures reduced physical contact among people (“social distancing”) and triggered unhealthier lifestyle habits, such as reduced levels of physical activity and unhealthy eating [1]. Not only did people change their lifestyle habits; many people also experienced a mental burden from the pandemic’s consequences for everyday life [2].

According to a meta-analysis of 74 studies, the prevalence of obesity increased both in adults and in children during the COVID-19 pandemic [3]. Across all adult participants who were included in the analysis (regardless of whether they gained, lost or maintained their weight), there was a mean body weight increase of 0.93 kg. Other studies have looked into the mechanisms behind pandemic-related weight gain and have identified reduced physical activity, unhealthy eating and mental stress as main drivers, among other associated factors like age, gender, education level and body mass index (BMI) [4, 5].

Given the increased risk that sustained weight gain brings for the development of chronic non-communicable diseases [6], it was alarming that the first year of the pandemic sparked many new cases of overweight and obesity. However, it is not well known whether these altered lifestyle habits in combination with weight gain persisted in later years or, over time, subsided [1]. Studies on this topic exist, but to our knowledge only covered the first year of the pandemic [7–10].

In two previous nation-wide representative surveys, we analyzed what impact the pandemic had on the lifestyles of children and adolescents in Germany and found unfavorable changes both in the first and the third year of the pandemic [11, 12]. These changes were most pronounced in children from families with low household incomes and in children with pre-existing overweight.

In the current study, we investigated the impact of the COVID-19 pandemic on lifestyle and body weight in adults in Germany, both directly after the first and after the second year of the pandemic. By means of two representative surveys, we analyzed to what extent changes seen in the first year of the pandemic continued to exist one year later. Furthermore, we investigated the association of health-related behaviors, mental stress and different sociodemographic factors with weight gain.

## Methods

We performed representative cross-sectional online surveys on lifestyle and body weight changes among German citizens aged 18 to 70 years at two different time periods during the COVID-19 pandemic: survey 1 (S1) from April 12th to 27th, 2021, and survey 2 (S2) from May 25th to June 2nd, 2022.

At the time of S1, in April 2021, the lockdowns that had taken effect initially in March 2020 and, after mitigation during the summer months, again in November 2020 had just been extended, as infection numbers were rising again. Restrictions imposed by the national and federal state governments to reduce both private and professional face-to-face contacts included the urgent request to work from home when possible, the urgent request not to travel and a limit to the number of people one could meet: social activities could only take place within one’s own household or with a total of five people from two households. Gyms were closed as well as restaurants, theatres and other recreational facilities. Citizens were asked to keep 1.5 m distance, to wear a medical face mask in public places and to stay home in case of symptoms typical of COVID-19 infection.

At the time of S2, in May and June 2022, most restrictions had been lifted. As of late March 2022 – after another winter lockdown – restaurants, bars, hotels, clubs and fitness studios had reopened. Large-scale events could take place again as well, provided that visitors were vaccinated or had recently recovered from COVID-19 infection. The requirement to wear a facemask stayed in effect only in indoor public places, and the 1.5-m distance rule had largely been revoked.

Survey participants (age range: 18 to 70 years) were recruited by the forsa Institute for Social Research and Statistical Analysis (forsa Politik- und Sozialforschung GmbH, Berlin, Germany). They were randomly selected from around 10,000 households in Germany listed in a panel representative for German speaking persons aged 14 years or older (forsa.omninet) [13]. This panel includes both people with a landline phone and people with a mobile phone, recruited per telephone through the ADM Sampling System [14]. All survey participants had access to an internet connection (i.e. at home, at work, at school, or through friends or family) and completed the survey online. They were recruited regardless of the frequency of their internet use. No participants in the surveys belonged to the same household. The study cohorts of S1 (*N* = 1,001) and S2 (*N* = 1,005) were independent of one another. The response rate was 40.1% for S1 and 33.5% for S2.

The questionnaire is available in Supplementary File 1. It primarily focused on changes in body weight, changes in dietary habits and changes in physical activity compared to before the pandemic. In S1, participants were asked to report changes since the beginning of the pandemic; in S2, participants were asked to report changes by comparing their current or past-year situation with their situation before the pandemic. In addition, participants were asked whether they experienced increased mental stress caused by the pandemic. Standard questions on demographic and socioeconomic characteristics were included as well. All data were self-reported.

To optimize the study’s validity for the general population of Germany, the collected data were weighted based on region of residence in combination with gender and age (region of residence x gender x age: 18 to 29 years, 30 to 44 years, 45 to 59 years, 60 to 70 years), based on the 16 federal states and according to education (general, intermediate, high, other), using data from the Federal Statistical Office as a reference. Weights were calculated by iterative proportional fitting [15] and were used in all descriptive and statistical analyses.

### Statistical analyses

Data are presented in the form of percentages of the respective sample and confidence intervals thereof. Continuous data are reported in the form of their means and standard deviations (SD).

As a variety of questions was asked regarding dietary habits, we mainly focused on the following: 1) one question on whether the participants were eating healthier or unhealthier than before the COVID-19 pandemic (referred to as the variable ‘healthy nutrition’, and 2) four questions on the consumption of energy-dense food, combined into a single variable ‘energy-dense food consumption’. The latter variable contained, like the original food groups (sweets, cakes and pastries, crisps and fast food), three subgroups: ‘less’, ‘no mean change’ and ‘more’ compared to before the COVID-19 pandemic. Taking all four food groups together, a score was calculated based on the respective counts of ‘less’ answers and ‘more’ answers, and if one of these answers dominated, the participant was categorized into the corresponding group; if not, the participant was categorized into ‘no mean change’.

Frequency tables were made of the outcome variables body weight (divided into ‘weight gain’ and ‘no change/weight loss’), physical activity (divided into ‘reduced’ and ‘no change/increased’), mental stress (divided into ‘not at all/mild’ and ‘moderate/severe’), healthy nutrition (divided into ‘less healthy’ and ‘no change/more healthy’) and energy-dense food group consumption (divided into ‘more’ and ‘no mean change/less’), stratified by survey and – per survey – stratified by baseline variables (gender, age, BMI, secondary education level and net household income). Furthermore, per survey, frequency tables were made of the outcome variables body weight, physical activity, healthy nutrition and energy-dense food group consumption stratified by mental stress levels.

Chi-square tests were performed on all frequency tables, including post-hoc pairwise Chi-square tests with Holm adjustment of p-values for variables consisting of more than two categories. Dietary behavior variables other than healthy nutrition and energy-dense food group consumption are presented in a descriptive manner without statistical tests.

For the primary outcome variable body weight, binomial logistic regression was performed to investigate the associations identified by the Chi-square tests. Adjusted odds ratios and p-values were calculated per subgroup with respect to the subgroup ‘no change’, or – if there was no category ‘no change’ – with respect to the subgroup with the lowest weight gain frequency as a reference. Before inclusion into the regression model, all variables were tested for multicollinearity by calculating variance inflation factors (VIF). A VIF of > 5 was considered to indicate multicollinearity. Because of the exploratory nature of the associations, no correction for multiple testing was performed. Statistical testing was performed in SPSS Statistics version 29 (IBM, Armonk, New York, USA), using the SPSS Complex Samples module to account for the sample weighting we performed. The sample weights were also taken into account in the calculation of confidence intervals, using the SPSS Complex Samples module. Figures were created using the pandas and matplotlib packages of Python version 3.11.3 (Python Software Foundation, Wilmington, Delaware, USA).

## Results

### Participant characteristics

The first survey was completed by 1,001 adults (mean age 45.4 ± 14.4 years), of whom 505 were men and 496 were women. The second survey was completed by 1,005 adults (mean age 45.7 ± 14.5 years), of whom 506 were men and 499 were women (Table 1). Mean BMI was 27.4 ± 6.0 kg/m^2^ in S1 and 27.1 ± 5.5 kg/m^2^ in S2 (Table 1).

**Table 1:** Characteristics of the survey participants

	S1 (*N* = 1,001) n (%) or mean ± SD		S2 (*N* = 1,005) **n (%) or mean ± SD**	
	Unweighted	Weighted	Unweighted	Weighted
**Gender**				
Men	479 (47.9%)	505 (50.4%)	524 (52.1%)	506 (50.4%)
Women	522 (52.1%)	496 (49.6%)	481 (47.9%)	499 (49.6%)
**Age (years)** ^a^	48.0 ± 13.4	45.4 ± 14.4	49.1 ± 13.3	45.7 ± 14.5
18-29	123 (12.3%)	198 (19.8%)	109 (10.8%)	195 (19.4%)
30-44	247 (24.7%)	275 (27.5%)	245 (24.4%)	281 (27.9%)
45-59	419 (41.9%)	328 (32.8%)	408 (40.6%)	324 (32.2%)
60-70	212 (21.2%)	200 (20.0%)	243 (24.2%)	205 (20.4%)
**Height (cm)**	173.9 ± 9.7	174.1 ± 9.7	174.0 ± 9.6	173.9 ± 9.4
**Weight (kg)** ^a^	84.0 ± 21.0	83.5 ± 20.7	83.1 ± 19.2	82.4 ± 19.1
**BMI (kg/m**^2^) ^a,b^	27.6 ± 6.1	27.4 ± 6.0	27.2 ± 5.5	27.1 ± 5.5
<25 (under-/normalweight)	349 (38.0%)	356 (38.7%)	371 (39.4%)	382 (41.0%)
25 to <30 (overweight)	321 (34.9%)	329 (35.7%)	326 (34.6%)	318 (34.2%)
≥30 (obese)	249 (27.1%)	235 (25.6%)	245 (26.0%)	231 (24.8%)
**Partner in household** ^b^				
Yes	654 (65.7%)	624 (62.5%)	670 (66.9%)	628 (62.9%)
No	342 (34.3%)	374 (37.5%)	331 (33.1%)	371 (37.1%)
**Children under 18 in household** ^b^				
Yes	287 (28.9%)	284 (28.6%)	264 (26.5%)	259 (26.0%)
No	706 (71.1%)	710 (71.4%)	731 (73.5%)	738 (74.0%)
**Secondary education level** ^b^				
General	249 (26.1%)	260 (27.1%)	248 (25.6%)	237 (24.6%)
Intermediate	337 (35.3%)	324 (33.7%)	347 (35.8%)	329 (34.0%)
High	368 (38.6%)	377 (39.2%)	373 (38.5%)	401 (41.4%)
**Net household income (euros)** ^b^				
<2,000	187 (21.2%)	198 (22.2%)	168 (18.1%)	183 (19.8%)
2,000 to <4,000	392 (44.2%)	401 (45.2%)	436 (47.1%)	423 (45.7%)
≥4,000	308 (34.7%)	289 (32.6%)	322 (34.8%)	319 (34.5%)

^a^ Not normally distributed according to histograms and Kolmogorov-Smirnov tests

^b^ Different sample sizes because of missing answers

*Abbreviations*: *BMI *body mass index

The percentage of people that stated they had changed jobs was 4% in S1 and 8% in S2. Job loss was in both surveys reported by 3% of the participants. 10% of the participants in S1, and 7% in S2 reported that their working hours had been temporarily reduced. Partial or permanent home office was reported by 25% of participants in S1 and 23% in S2. 49% (S1) and 50% (S2) of the participants answered that their employment status had not changed.

### Pandemic-related lifestyle changes

#### Mental stress

**Fig. 1:**
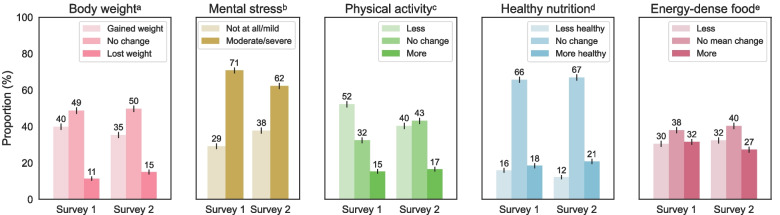
Reported changes in body weight, mental stress levels and physical activity levels compared to before the COVID-19 pandemic, expressed as proportions (%) of survey participants. 95%-confidence intervals are indicated by black vertical lines. **a** Survey 1, 2: ‘Has your body weight changed since the beginning of the COVID-19 pandemic?’ (answer options: yes, I gained weight; yes, I lost weight; no; don’t know/no comment). **b** Survey 1: ‘How strongly do you feel mentally stressed by the changes in relation to the COVID-19 situation?’ (answer options: not at all; mildly; moderately; severely; don’t know/no comment), survey 2: ‘How strongly did you, in the past year, feel mentally stressed by the changes in relation to the COVID-19 situation?’ (answer options: not at all; mildly; moderately; severely; don’t know/no comment). ‘Not at all’ and ‘mildly’ answers were taken together into ‘not at all/mild’; ‘moderately’ and ‘severely’ answers were taken together into ‘moderate/severe’. **c** Survey 1: ‘Would you say you, since the beginning of the COVID-19 pandemic, altogether moved more or less or was there no substantial change?’ (answer options: more than before; less than before; no change; don’t know/no comment), survey 2: ‘Would you say you, in the past year, altogether moved more or less or was there no substantial change compared to the situation before the COVID-19 pandemic?’ (answer options: more than before; less than before; no change; don’t know/no comment). **d** Survey 1: ‘In what way has your dietary behavior changed since the beginning of the pandemic?’ (answer options: I eat healthier; I eat less healthy; no substantial change; don’t know/no comment), survey 2: ‘In what way has your dietary behavior changed compared to before the COVID-19 pandemic?’ (answer options: I eat healthier; I eat less healthy; no substantial change; don’t know/no comment). **e** Survey 1: ‘Please indicate whether you, since the beginning of the COVID-19 pandemic, have eaten more, less or equal amounts of the following food groups.’ (answer options: more; less; equal amounts; don’t know/no comment), survey 2: ‘Please indicate whether you, in the past year compared to before the COVID-19 pandemic, have eaten more, less or equal amounts of the following food groups.’ (answer options: more; less; equal amounts; don’t know/no comment). ‘Don’t know/no comment’ answers are not shown and were not considered in the proportion calculations

A vast majority of the participants reported to have felt moderately or severely stressed because of the COVID-19 pandemic: 71% in S1 and 62% in S2 (*p* < 0.001, Supplementary Table 1; Fig. 1). The remaining 29% (S1) and 38% of people (S2) had been feeling mildly stressed or not stressed at all.

Frequency tables and statistical results of mental stress levels stratified by gender, age, BMI, secondary education level and net household income can be found in Supplementary Table 2. Women more frequently felt moderately or severely stressed than men (77% vs. 65% in S1, *p* < 0.001; 70% vs. 55% in S2, *p* < 0.001). In S2, less people felt moderately or severely stressed in the age group of 60–70 years (49%) compared to 71% and 68% in the age groups of 18–29 years (*p* < 0.001) and 30–44 years (*p* < 0.001), respectively. A difference in mental stress levels was observed between participants aged 45–59 years (60% moderately or severely stressed in S2) and participants aged 18–29 years (71% moderately or severely stressed in S2, *p* = 0.039). Furthermore, in S2, individuals with a BMI of ≥ 30 kg/m^2^ more frequently reported moderate to severe stress (71%) than those with a BMI of < 25 kg/m^2^ (61%, *p* = 0.021) and those with a BMI of 25 to < 30 kg/m^2^ (57%,* p* = 0.001). In addition, mental stress levels were higher in participants with a high education level (71%) compared to 57% and 53% in participants with a general (*p* = 0.002) and intermediate education level (*p* < 0.001), respectively. These associations with age, BMI and education level were not observed in S1.

#### Physical activity

In S1, 52% of the participants reported to have been less physically active during the pandemic compared to the situation before (Fig. 1). This percentage was lower in S2: 40% (*p* < 0.001, Supplementary Table 1).

In S1, reduced physical activity was more often reported by those with a high secondary education level (60%) compared to those with a general (47%, *p* = 0.003) or an intermediate (50%, *p* = 0.011) education level, and more often by those with a BMI of ≥ 30 kg/m^2^ (60%) than by those with a BMI of < 25 kg/m^2^ (49%,* p* = 0.022; Supplementary Table 3). Such associations were also observed in S2: reduced physical activity levels were more frequently reported by participants with a high education level (48%) than by those with a general (35%, *p* = 0.002) or an intermediate education level (33%, *p* < 0.001), and more frequently by participants with a BMI of ≥ 30 kg/m^2^ (49%) than by those with a BMI of < 25 kg/m^2^ (35%, *p* = 0.002) or a BMI of 25 to < 30 kg/m^2^ (39%, *p* = 0.036; Supplementary Table 3). Furthermore, in S2, differences were observed between genders (45% of women reported reduced physical activity compared to 36% of men, *p* = 0.007). Also, reduced physical activity was less often reported by participants aged 60–70 years (27%) than by those aged 18–29 years (47%, *p* < 0.001), 30–44 years (48%, *p* < 0.001) and 45–59 years in S2 (38%, *p* = 0.038).

Mental stress levels were also associated with reductions in physical activity (Supplementary Table 6). Of the people with moderate to severe stress in S1 (*n* = 707), 56% reported reduced physical activity, compared to 43% of the people with mild or no stress (*p* = 0.001). In S2, 50% of all people with moderate to severe stress (*n* = 626) reported reduced physical activity, compared to 25% of the people with mild or no stress (*p* < 0.001).

Participants who reported to have moved less, often mentioned the closure of gyms and other indoor sports facilities as a main reason (53% and 40% in S1 and S2, respectively; data not shown). Similarly, the limited offer of private or group-based sports sessions was reported as a reason (by 33% and 29%, respectively; data not shown). Reductions in physical activity were work-related in 31% (both surveys), motivation-related in 31% and 34%, respectively, and time-related in 10% and 17% of the participants, respectively (data not shown).

#### Dietary habits

The percentage of people who had changed to a less healthy diet decreased from 16% in S1 to 12% in S2 (*p* = 0.033, Supplementary Table 1, Fig. 1). Frequencies of the variable ‘healthy nutrition’ stratified for demographic and socioeconomic variables can be found in Supplementary Table 4. In S1, participants aged 60–70 years less frequently (7%) reported unhealthier eating than the other age groups (18–29 years: 18%, *p* = 0.007; 30–44 years: 21%, *p* < 0.001; 45–59 years: 15%, *p* = 0.028). Persons with a BMI of ≥ 30 kg/m^2^ more frequently reported (22%) unhealthier eating than persons with a BMI of < 25 kg/m^2^ (13%, *p* = 0.009) or with a BMI of 25 to < 30 kg/m^2^ in S1 (14%, *p* = 0.030). In S2, unhealthier eating was dependent on age and BMI as well: differences were observed between the age groups of 18–29 years and 60–70 years (15% and 6%, *p* = 0.020), 30–44 years and 45–59 years (17% and 10%, *p* = 0.036), and 30–44 years and 60–70 years (17% and 6%, *p* = 0.002). Participants with a BMI of < 25 kg/m^2^ less frequently (6%) reported unhealthier eating than those with a BMI of 25 to < 30 kg/m^2^ (14%, *p* = 0.002) or with a BMI of ≥ 30 kg/m^2^ (17%, *p* < 0.001) in S2. Furthermore, a difference was observed between participants with a net household income of ≥ 4,000 euros, of whom 17% reported unhealthier eating, and those with an income of 2,000 to < 4,000 euros in S2 (9%, *p* = 0.026).

Survey results of further dietary habits are shown in Table 2. In S1, 34% of people reported that they had more time to eat. In S2, this percentage was 24%. Furthermore, 28% (S1) and 21% (S2) reported that they ate more often out of boredom. The percentage of people who had used food more frequently as a reward was 18% in S1 and 14% in S2.

**Table 2:** Changes in dietary habits, food group consumption and food purchase and preparation compared to before the COVID-19 pandemic, expressed as proportions (%) of the entire sample

	S1 (*N* = 1,001)			S2 (*N* = 1,005)		
	**Less**	**No change**	**More**	**Less**	**No change**	**More**
	**% [95%-CI]**	**% [95%-CI]**	**% [95%-CI]**	**% [95%-CI]**	**% [95%-CI]**	**% [95%-CI]**
**Dietary habits** ^a^						
Eating healthy ^b^	16 [14, 18]	66 [62, 69]	18 [16, 21]	12 [10, 15]	67 [64, 70]	21 [18, 24]
Eating regularly ^c^	18 [15, 21]	67 [64, 70]	15 [13, 17]	16 [13, 19]	72 [69, 75]	12 [10, 15]
Eating main meals ^d^	7.4 [5.8, 9.4]	82 [79, 85]	10 [8.5, 13]	10 [7.8, 12]	84 [81, 86]	6.8 [5.2, 8.9]
Eating snacks ^e^	6.6 [5.2, 8.5]	67 [64, 70]	27 [24, 30]	9.2 [7.3, 11]	69 [66, 73]	21 [19, 24]
Portion size ^f^	8.3 [6.6, 10]	81 [78, 84]	11 [8.7, 13]	11 [8.8, 13]	79 [76, 82]	10 [8.2, 12]
Appetite ^g^	8.6 [6.9, 11]	68 [65, 71]	23 [21, 26]	8.4 [6.7, 11]	73 [70, 76]	19 [16, 22]
Time to eat ^h^	5.5 [4.1, 7.2]	61 [58, 64]	34 [30, 37]	5.8 [4.5, 7.6]	71 [67, 74]	24 [21, 27]
Eating out of boredom ^i^	5.6 [4.2, 7.3]	66 [63, 69]	28 [25, 31]	8.2 [6.5, 10]	71 [68, 74]	21 [18, 24]
Using food as a reward ^j^	4.6 [3.4, 6.2]	77 [74, 80]	18 [16, 21]	4.9 [3.6, 6.5]	81 [78, 83]	14 [12, 17]
**Food group consumption** ^k^						
Sweets	12 [10, 14]	58 [55, 62]	30 [27, 33]	13 [11, 15]	64 [61, 67]	23 [20, 26]
Cakes and pastries	15 [13, 18]	60 [57, 63]	25 [22, 28]	17 [14, 20]	65 [62, 68]	18 [16, 21]
Crisps	18 [15, 21]	62 [58, 65]	20 [17, 23]	20 [18, 23]	64 [60, 67]	16 [14, 19]
Fast food	36 [32, 39]	48 [45, 52]	16 [14, 19]	33 [30, 36]	54 [51, 58]	13 [11, 16]
Fruits	11 [9.5, 14]	67 [64, 70]	21 [19, 24]	9.4 [7.6, 12]	68 [65, 71]	22 [20, 25]
Vegetables	7.1 [5.6, 8.9]	69 [65, 72]	24 [22, 27]	5.7 [4.3, 7.4]	69 [66, 72]	26 [23, 29]
Alcoholic beverages	29 [25, 32]	55 [51, 58]	17 [14, 20]	30 [26, 33]	55 [51, 58]	16 [13, 19]
Coffee	10 [7.7, 12]	67 [63, 70]	24 [21, 27]	10 [7.7, 12]	69 [66, 73]	21 [18, 24]
Soft drinks	19 [17, 22]	71 [67, 74]	10 [8.0, 12]	24 [21, 27]	67 [63, 70]	9.4 [7.5, 12]
**Food purchase and preparation** ^l^						
Cooking/preparing meals	6.2 [4.8, 8.0]	60 [56, 63]	34 [31, 38]	6.7 [5.2, 8.6]	60 [57, 64]	33 [30, 36]
Ordering meal delivery	18 [16, 21]	54 [50, 57]	28 [25, 31]	18 [15, 20]	57 [53, 60]	26 [23, 29]
Getting take-out meals	29 [26, 32]	41 [38, 45]	29 [26, 33]	25 [22, 28]	50 [47, 54]	25 [22, 28]
Grocery shopping	19 [16, 22]	59 [55, 62]	22 [20, 25]	13 [11, 16]	70 [66, 73]	17 [15, 20]

‘Don’t know/no comment’ answers are not shown and were not considered in the proportion calculations

^a^Survey 1: ‘In what way has your dietary behavior changed since the beginning of the pandemic?’, survey 2: ‘In what way has your dietary behavior changed compared to before the COVID-19 pandemic?’

^b^Answer options: I eat healthier; I eat less healthy; no substantial change; don’t know/no comment

^c^Answer options: I eat more regularly; I eat less regularly; no substantial change; don’t know/no comment

^d^Answer options: I eat more main meals; I eat fewer main meals; no substantial change; don’t know/no comment

^e^Answer options: I eat more snacks; I eat fewer snacks; no substantial change; don’t know/no comment

^f^Answer options: I eat larger portions; I eat smaller portions; no substantial change; don’t know/no comment

^g^Answer options: I have an increased appetite; I have a decreased appetite; no substantial change; don’t know/no comment

^h^Answer options: I have less time to eat; I have more time to eat; no substantial change; don’t know/no comment

^i^Answer options: I eat more often out of boredom; I eat less often out of boredom; no substantial change; don’t know/no comment

^j^Answer options: I use food more often as a reward; I use food less often as a reward; no substantial change; don’t know/no comment

^k^Survey 1: ‘Please indicate whether you, since the beginning of the COVID-19 pandemic, have eaten more, less or equal amounts of the following food groups.’ (answer options: more; less; equal amounts; don’t know/no comment), survey 2: ‘Please indicate whether you, in the past year compared to before the COVID-19 pandemic, have eaten more, less or equal amounts of the following food groups.’ (answer options: more; less; equal amounts; don’t know/no comment)

^l^Survey 1: ‘Please indicate whether you, since the beginning of the COVID-19 pandemic, have done the following things more often, less often or just as often as before.’ (answer options: more often; less often; just as often/no change; don’t know/no comment), survey 2: ‘Please indicate whether you, in the past year compared to before the COVID-19 pandemic, have done the following things more often, less often or just as often as before.’ (answer options: more often; less often; just as often/no change; don’t know/no comment)

In both surveys, less healthy eating was more often reported by participants with moderate to severe stress than by those with no or mild stress (20% vs 6% in S1, *p* < 0.001; 16% vs 7% in S2, *p* < 0.001; Supplementary Table 6).

#### Food group consumption

Increased consumption of energy-dense food groups (sweets, cakes and pastries, crisps and fast food) was reported by 32% of the participants in S1 and 27% in S2 (*p* = 0.060, Supplementary Table 1, Fig. 1). In both surveys, the age group of 60–70 years less frequently reported increased consumption of these food groups (16% in S1 and 14% in S2) than all other age groups (18–29 years: 33% in S1 and 35% in S2; 30–44 years: 41% in S1 and 37% in S2; 45–59 years: 32% in S1 and 23% in S2; *p*-values in Supplementary Table 5). In S1, participants with a BMI of ≥ 30 kg/m^2^ more frequently (42%) reported increased consumption of energy-dense food than those with a BMI of < 25 kg/m^2^ (27%, *p* < 0.001) or a BMI of 25 to < 30 kg/m^2^ (29%, *p* = 0.002). Furthermore, differences were observed between a high education level (37% in S1 and 35% in S2) and a general education level (26% in S1, *p* = 0.016; 22% in S2, *p* = 0.002), and – only in S2 – between a high education level (35%) and an intermediate education level (22%, *p* < 0.001). Finally, in S2, participants with a net household income of ≥ 4,000 euros more frequently (37%) reported increased consumption of energy-dense food than those with a net household income of < 2,000 euros (20%, *p* < 0.001) or a net household income of 2,000 to < 4,000 euros (23%, *p* < 0.001; Supplementary Table 5).

With regard to the consumption of fruits, 21% (S1) and 22% of people (S2) reported an increase, whereas 11% (S1) and 9% (S2) reported a decrease (Table 2). An increased consumption of vegetables was reported by 24% (S1) and 26% of people (S2); a decreased consumption of vegetables was reported by 7% (S1) and 6% (S2). Results for the increased and decreased consumption of coffee and alcoholic beverages and soft drinks are shown in Table 2.

It is noteworthy that participants with moderate to severe mental stress (*n* = 707 in S1, *n* = 626 in S2) more often reported increased consumption of energy-dense food groups than participants with mild or no stress (S1: 36% vs 20%, *p* < 0.001; S2: 34% vs 16%, *p* < 0.001; Supplementary Table 6). Increased consumption of fast food, alcoholic beverages and coffee was reported by 18%, 19% and 26% (S1) and 16%, 21% and 25% (S2) of people with moderate to severe stress, and by 9%, 10% and 17% (S1) and 8%, 8% and 15% (S2) of people with mild or no stress (data not shown). Decreased consumption of fruits was reported by 14.0% (S1) and 12.1% (S2) of individuals with moderate to severe stress, and by 5.3% (S1) and 5.0% (S2) of individuals with mild or no stress (data not shown). Decreased consumption of vegetables was reported by 8.7% (S1) and 7.6% (S2) of individuals with moderate to severe stress, and by 3.1% (S1) and 2.4% (S2) of individuals with mild or no stress (data not shown).

#### Food purchase and preparation

In both surveys, approximately one-third of the participants reported that they had been cooking and preparing meals more frequently than before the pandemic (Table 2). 6% (S1) and 7% (S2) of the participants reported less frequent cooking and preparing meals. An increase in getting take-out meals was reported by 29% (S1) and 25% (S2) of the participants (Table 2). Similar percentages of people reported a decrease in getting take-out meals (29% in S1 and 25% in S2). Finally, 28% (S1) and 26% (S2) of the participants stated they had more frequently ordered meals from home delivery services; 18% (S1 and S2) stated they had done this less frequently (Table 2).

### Pandemic-related body weight changes

#### Weight gain

In S1, 40% of all participants answered that they had gained weight (Fig. 1). In S2, this percentage was 35% (*p* = 0.059, Supplementary Table 1). On average, the reported weight gain among those who gained weight was 5.5 ± 3.3 kg in S1 and 6.5 ± 4.4 kg in S2.

**Fig. 2:**
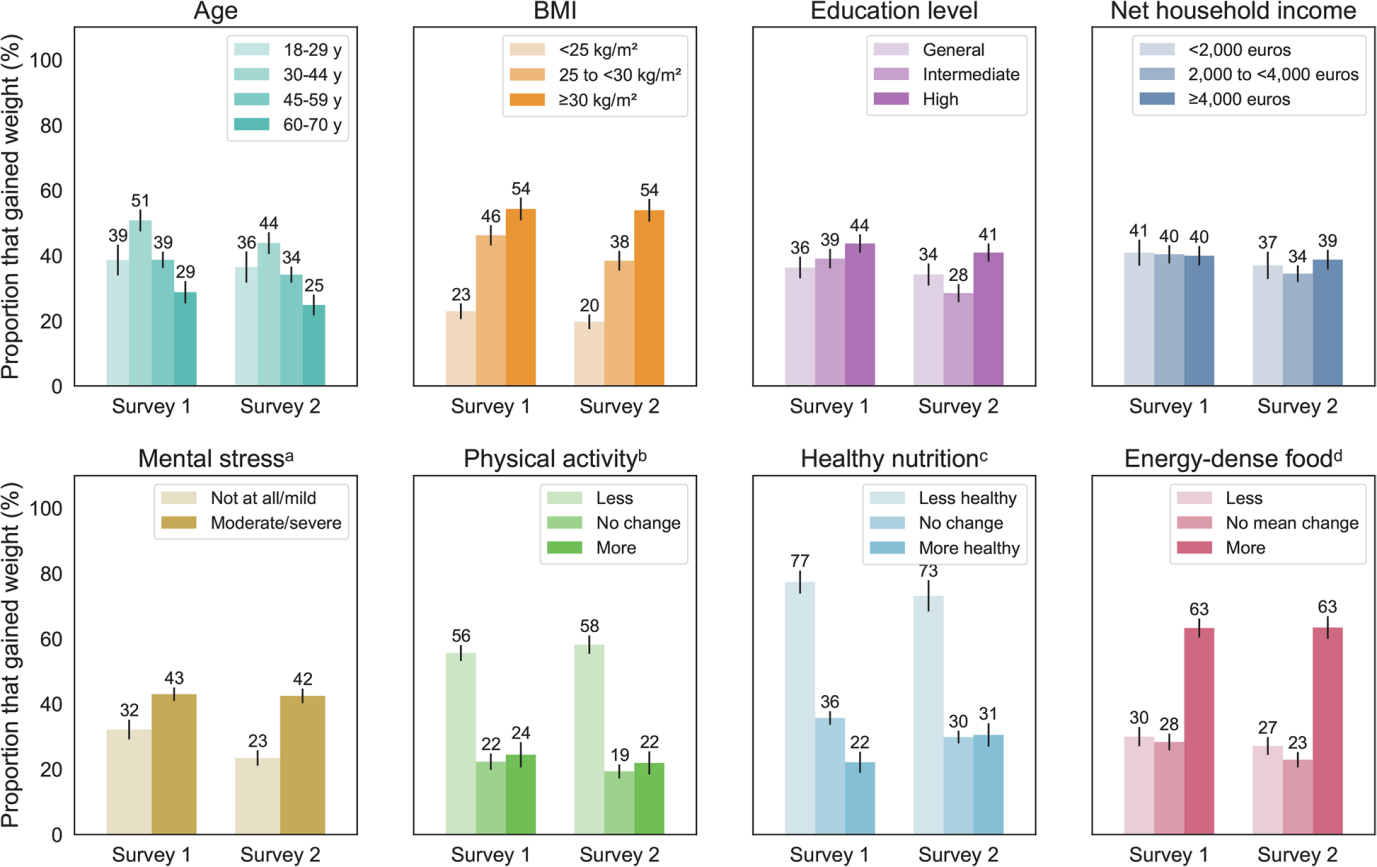
Proportions (%) of survey participants that gained weight broken down into subgroups of age, BMI at the time of the survey, education level, net household income, mental stress, changes in physical activity, healthier/unhealthier nutrition, and consumption of energy-dense food (sweets, cakes and pastries, crisps and fast food taken together). Each plot accommodates the results of both S1 and S2. 95%-confidence intervals are indicated by black vertical lines. Abbreviations: y, years; BMI, body mass index. **a** Survey 1: ‘How strongly do you feel mentally stressed by the changes in relation to the COVID-19 situation?’ (answer options: not at all; mildly; moderately; severely; don’t know/no comment), survey 2: ‘How strongly did you, in the past year, feel mentally stressed by the changes in relation to the COVID-19 situation?’ (answer options: not at all; mildly; moderately; severely; don’t know/no comment). ‘Not at all’ and ‘mildly’ answers were taken together into ‘not at all/mild’; ‘moderately’ and ‘severely’ answers were taken together into ‘moderate/severe’. **b** Survey 1: ‘Would you say you, since the beginning of the COVID-19 pandemic, altogether moved more or less or was there no substantial change?’ (answer options: more than before; less than before; no change; don’t know/no comment), survey 2: ‘Would you say you, in the past year, altogether moved more or less or was there no substantial change compared to the situation before the COVID-19 pandemic?’ (answer options: more than before; less than before; no change; don’t know/no comment). **c** Survey 1: ‘In what way has your dietary behavior changed since the beginning of the pandemic?’ (answer options: I eat healthier; I eat less healthy; no substantial change; don’t know/no comment), survey 2: ‘In what way has your dietary behavior changed compared to before the COVID-19 pandemic?’ (answer options: I eat healthier; I eat less healthy; no substantial change; don’t know/no comment). **d** Survey 1: ‘Please indicate whether you, since the beginning of the COVID-19 pandemic, have eaten more, less or equal amounts of the following food groups.’ (answer options: more; less; equal amounts; don’t know/no comment), survey 2: ‘Please indicate whether you, in the past year compared to before the COVID-19 pandemic, have eaten more, less or equal amounts of the following food groups.’ (answer options: more; less; equal amounts; don’t know/no comment). ‘Don’t know/no comment’ answers are not shown and were not considered in the proportion calculations

To investigate which population groups were most prone to weight gain, we investigated various sociodemographic factors, mental stress levels and health-related behaviors. Chi-square test results are shown in Table 3. Considering subgroups according to ‘weight gain’ and ‘no weight change/weight loss’, statistically significant associations (*p* < 0.05) were observed of weight gain frequency with age, BMI at the time of the survey, mental stress, physical activity, and dietary changes with regard to the healthiness of the participants’ diets and their consumption of energy-dense food. Education level was in S2 significantly associated with weight gain frequency (Table 3). In Fig. 2, weight gain proportions are presented per variable (with the exception of the variables ‘gender’ and ‘partner in household’, for which the Chi-square tests did not indicate an association with weight gain).

**Table 3:** Proportions of survey participants reporting weight gain versus no change/weight loss, broken down into subgroups based on demographic and socioeconomic factors, mental stress and health-related behaviors. Chi-square test results are included in the form of *p*-values, with *p*-values of < 0.05 (displayed in bold) indicating a significant association between the variable and weight change status

	**S1 (** ***N*** ** = 1,001)**			**S2 (** ***N*** ** = 1,005)**		
	**Gained weight**	**No change/lost weight**		**Gained weight**	**No change/lost weight**	
	**% [95%-CI]**	**% [95%-CI]**	***p***	**% [95%-CI]**	**% [95%-CI]**	***p***
**Total**	40 [37, 43]	60 [57, 63]		35 [32, 39]	65 [61, 68]	
**Gender**						
Men	40 [35, 45]	60 [55, 65]	0.948	35 [31, 40]	65 [60, 69]	0.849
Women	40 [35, 45]	60 [55, 65]		36 [31, 41]	64 [59, 69]	
**Age (years)**						
18–29	39 [30, 48]	61 [52, 70]	**< .001**	36 [28, 46]	64 [54, 72]	**0.003**
30–44	51 [44, 57]	49 [43, 56]		44 [37, 50]	56 [50, 63]	
45–59	39 [34, 44]	61 [56, 66]		34 [29, 39]	66 [61, 71]	
60–70	29 [23, 36]	71 [64, 77]		25 [19, 32]	75 [68, 81]	
**BMI (kg/m**^**2**^**)**						
< 25 (under-/normalweight)	23 [19, 28]	77 [72, 81]	**< .001**	20 [16, 24]	80 [76, 84]	**< .001**
25 to < 30 (overweight)	46 [40, 52]	54 [48, 60]		38 [33, 44]	62 [56, 67]	
≥ 30 (obese)	54 [47, 61]	46 [39, 53]		54 [47, 61]	46 [39, 53]	
**Partner in household**						
Yes	41 [37, 45]	59 [55, 63]	0.584	36 [32, 40]	64 [60, 68]	0.613
No	39 [33, 45]	61 [55, 67]		34 [29, 40]	66 [60, 71]	
**Secondary education level**						
General	36 [30, 43]	64 [57, 70]	0.222	34 [28, 41]	66 [59, 72]	**0.007**
Intermediate	39 [33, 45]	61 [55, 67]		28 [23, 34]	72 [66, 77]	
High	44 [38, 49]	56 [51, 62]		41 [36, 46]	59 [54, 64]	
**Net household income (euros)**						
< 2,000	41 [33, 49]	59 [51, 67]	0.982	37 [29, 45]	63 [55, 71]	0.561
2,000 to < 4,000	40 [35, 46]	60 [54, 65]		34 [30, 40]	66 [60, 70]	
≥ 4,000	40 [34, 46]	60 [54, 66]		39 [33, 45]	61 [55, 67]	
**Mental stress**						
Not at all/mildly stressed	32 [27, 38]	68 [62, 73]	**0.004**	23 [19, 28]	77 [72, 81]	**< .001**
Moderately/severely stressed	43 [39, 47]	57 [53, 61]		42 [38, 47]	58 [53, 62]	
**Physical activity**						
Less	56 [51, 60]	44 [40, 49]	**< .001**	58 [53, 64]	42 [36, 47]	**< .001**
No change	22 [18, 28]	78 [72, 82]		19 [15, 24]	81 [76, 85]	
More	24 [18, 33]	76 [67, 82]		22 [16, 30]	78 [70, 84]	
**Healthy nutrition**						
Eating less healthy	77 [70, 84]	23 [16, 30]	**< .001**	73 [63, 81]	27 [19, 37]	**< .001**
No change	36 [32, 40]	64 [60, 68]		30 [26, 34]	70 [66, 74]	
Eating more healthy	22 [16, 29]	78 [71, 84]		31 [24, 38]	69 [62, 76]	
**Energy-dense food consumption**						
Less	30 [25, 36]	70 [64, 75]	**< .001**	27 [22, 33]	73 [67, 78]	**< .001**
No mean change	28 [24, 34]	72 [66, 76]		23 [19, 28]	77 [72, 81]	
More	63 [57, 69]	37 [31, 43]		63 [56, 70]	37 [30, 44]	

*Abbreviations*: *95%-CI* 95%-confidence interval, *BMI* body mass index

Table 4 contains the results of the binomial logistic regression analysis and shows which variables were associated with weight gain. Based on the findings in the participants of S1, the odds for weight gain were significantly higher (*p* < 0.05) for ages of 33–44 years compared to ages above 59 years, BMI of ≥ 25 kg/m^2^ compared to BMI of < 25 kg/m^2^, reduced physical activity levels compared to no change, less healthy nutrition compared to no change, more healthy nutrition compared to no change, and increased consumption of energy-dense food groups compared to no mean change. The highest adjusted ORs (AOR) in S1 were observed in the following subgroups: BMI of ≥ 30 kg/m^2^ (AOR = 4.98), reduced physical activity (AOR = 3.26), less healthy nutrition (AOR = 3.22), increased consumption of energy-dense food (AOR = 2.24) and ages of 33–44 years (AOR = 2.10) (Table 4).

**Table 4:** Binomial logistic regression results of the variables that were identified to be significantly associated with weight change status (weight gain vs. no change/weight loss). *P*-values of < 0.05 are displayed in bold and indicate an association of the respective variable with weight change status

	**S1 (** ***N*** ** = 1,001)**		**S2 (N = 1,005)**	
	**Adj. OR [95%-CI]**	***p***	**Adj. OR [95%-CI]**	***p***
**Age (years)**		**0.015**		0.630
18–29	1.79 [0.93; 3.46]	0.084	1.27 [0.63; 2.57]	0.504
30–44	2.10 [1.23; 3.61]	**0.007**	1.43 [0.84; 2.44]	0.191
45–59	1.11 [0.69; 1.78]	0.657	1.18 [0.74; 1.87]	0.480
60–70	*reference*		*reference*	
**BMI (kg/m**^**2**^**)**		**< .001**		**< .001**
< 25 (under-/normalweight)	*reference*		*reference*	
25 to under 30 (overweight)	3.76 [2.42; 5.84]	**< .001**	3.23 [2.02; 5.16]	**< .001**
≥ 30 (obese)	4.98 [3.14; 7.88]	**< .001**	5.85 [3.47; 9.87]	**< .001**
**Mental stress**				
Not at all/mildly stressed	*reference*		*reference*	
Moderately/severely stressed	1.12 [0.77; 1.63]	0.565	1.37 [0.92; 2.04]	0.123
**Physical activity**		**< .001**		**< .001**
Reduced	3.26 [2.18; 4.89]	**< .001**	2.76 [1.83; 4.15]	**< .001**
No change	*reference*		*reference*	
Increased	1.11 [0.61; 2.03]	0.729	0.71 [0.41; 1.22]	0.210
**Secondary education level**		0.457		0.146
General	1.00 [0.62; 1.59]	0.984	1.25 [0.79; 1.99]	0.337
Intermediate	*reference*		*reference*	
High	1.27 [0.84; 1.91]	0.263	1.59 [1.00; 2.53]	**0.049**
**Healthy nutrition**		**< .001**		**0.025**
Eating less healthy	3.22 [1.90; 5.45]	**< .001**	2.47 [1.27; 4.80]	**0.008**
No change	*reference*		*reference*	
Eating more healthy	0.62 [0.38; 1.00]	**0.048**	1.22 [0.77; 1.92]	0.399
**Energy-dense food consumption**		**< .001**		**< .001**
Less	1.12 [0.72; 1.74]	0.613	1.19 [0.77; 1.85]	0.429
No mean change	*reference*		*reference*	
More	2.24 [1.45; 3.46]	**< .001**	3.61 [2.23; 5.83]	**< .001**

*Abbreviations*: *adj. OR* adjusted odds ratio, *95%-CI* 95%-confidence interval, *BMI* body mass index

In S2, similar findings were obtained, except that none of the age groups had significantly different odds for weight gain than the age group of 60–70 years. Furthermore, a high education level was found to have higher odds for weight gain than an intermediate education level. In S2, the highest adjusted ORs were observed in the following subgroups: BMI of ≥ 30 kg/m^2^ compared to BMI of < 25 kg/m^2^ (AOR = 5.85), increased consumption of energy-dense food compared to no mean change (AOR = 3.61), reduced physical activity compared to no change (AOR = 2.76), less healthy nutrition compared to no change (AOR = 2.47), and a high education level compared to an intermediate education level (AOR = 1.59) (Table 4).

#### Weight loss

The percentage of people reporting weight loss was 11% in S1 and 15% in S2 (Fig. 1). Average weight loss among those who lost weight was 6.4 ± 4.3 kg in S1 and 7.9 ± 6.6 kg in S2. Almost half of the participants – a total of 46% in both surveys – reported to have undertaken efforts to lose weight in the past six months (data not shown).

**Fig. 3:**
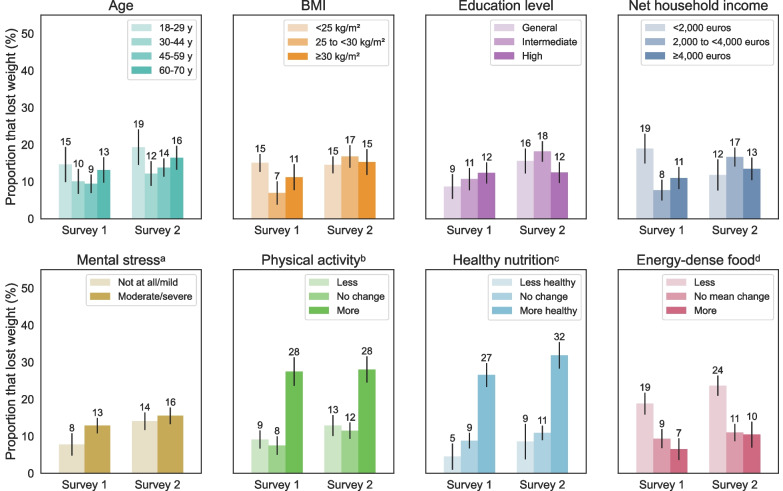
Proportions (%) of survey participants that lost weight broken down into subgroups of age, BMI at the time of the survey, education level, net household income, mental stress, changes in physical activity, healthier/unhealthier nutrition, and consumption of energy-dense food (sweets, cakes and pastries, crisps and fast food taken together). Each plot accommodates both the results of S1 and the results of S2. 95%-confidence intervals are indicated by black vertical lines. Abbreviations: y, years; BMI, body mass index. **a** Survey 1: ‘How strongly do you feel mentally stressed by the changes in relation to the COVID-19 situation?’ (answer options: not at all; mildly; moderately; severely; don’t know/no comment), survey 2: ‘How strongly did you, in the past year, feel mentally stressed by the changes in relation to the COVID-19 situation?’ (answer options: not at all; mildly; moderately; severely; don’t know/no comment). ‘Not at all’ and ‘mildly’ answers were taken together into ‘not at all/mild’; ‘moderately’ and ‘severely’ answers were taken together into ‘moderate/severe’. **b** Survey 1: ‘Would you say you, since the beginning of the COVID-19 pandemic, altogether moved more or less or was there no substantial change?’ (answer options: more than before; less than before; no change; don’t know/no comment), survey 2: ‘Would you say you, in the past year, altogether moved more or less or was there no substantial change compared to the situation before the COVID-19 pandemic?’ (answer options: more than before; less than before; no change; don’t know/no comment). **c** Survey 1: ‘In what way has your dietary behavior changed since the beginning of the COVID-19 pandemic?’ (answer options: I eat healthier; I eat less healthy; no substantial change; don’t know/no comment), survey 2: ‘In what way has your dietary behavior changed compared to before the COVID-19 pandemic?’ (answer options: I eat healthier; I eat less healthy; no substantial change; don’t know/no comment). **d** Survey 1: ‘Please indicate whether you, since the beginning of the COVID-19 pandemic, have eaten more, less or equal amounts of the following food groups.’ (answer options: more; less; equal amounts; don’t know/no comment), survey 2: ‘Please indicate whether you, in the past year compared to before the COVID-19 pandemic, have eaten more, less or equal amounts of the following food groups.’ (answer options: more; less; equal amounts; don’t know/no comment). ‘Don’t know/no comment’ answers are not shown and were not considered in the proportion calculations

In Fig. 3, findings regarding weight loss are shown. Age, changes in physical activity and dietary changes showed systematic patterns in the two surveys (which were in fact opposite to the patterns seen for weight gain). Also, the gender-specific results changed: in S1, 14% of women and 8% of men reported to have lost weight, whereas in S2, 15% of men as well as women stated to have lost weight.

## Discussion

Two online surveys embedded in a panel of adults in Germany revealed a complex picture of how individuals modified, and later partly readjusted their health-related behavior in response to the COVID-19 pandemic and the consecutive restrictions imposed by governmental authorities. We observed that weight gain was associated with higher BMI at the time of the survey, reduced physical activity and unhealthier dietary behavior.

Among the participants who reported changes in their dietary habits, there was heterogeneity from more conscious and healthy eating to increased consumption of energy-dense food. Whereas the results of S1 indicated that people were more likely to increase than decrease their consumption of energy-dense food, more balanced findings were obtained in S2, suggesting that some people managed to readjust their eating patterns.

The number of people reporting lower physical activity levels decreased, but remained substantial, although most sports facilities had reopened at the time of S2. This is an alarming finding considering the role of physical activity for general health and may suggest that people were still hesitant to use sports facilities and attend sports classes. A sense of normal life had apparently not yet returned.

Reductions in physical activity were more frequently reported in our study (52% in S1, 40% in S2) than in the GEDA 2021 study [16]. That study found that 24% of the people in Germany less frequently engaged in sports since the beginning of the pandemic. The results of the two studies are not directly comparable, due to a difference in phrasing and different timings of the surveys. The GEDA study collected data between July and October 2021 and our study was conducted in April 2021 (S1) and May and June 2022 (S2).

As a result of these diverse changes in lifestyle, 40% of people in S1 and 35% of people in S2 reported weight gain. The lower percentage in S2 is in line with the findings of the participants’ physical activity levels and dietary patterns. The observation that participants with a BMI of ≥ 30 kg/m^2^ more frequently gained weight agrees with another German study and a systematic review [4, 17]. The current study shows that this association continued to exist in the third year of the pandemic. It should be noted that the reported BMI was not pre-pandemic but BMI at the time of the survey. Hence, weight gain might have had an effect on the categorization of participants into the different BMI groups.

It is possible that weight stigma contributed to the observed association between obesity and weight gain [18, 19]. Weight stigma harms both the physical and mental wellbeing of people with obesity and triggers further weight gain [18]. In two position statements, the European Association for the Study of Obesity (EASO) stressed stigmatization and discrimination of people with obesity as problems in providing adequate care to this population during the COVID-19 pandemic [20, 21]. This is concerning because obesity is a risk factor for intensive care unit admissions and respiratory failure caused by COVID-19 [22, 23].

The participants included in our surveys gained weight more frequently than to be expected from proportions measured globally. Mekanna and co-authors included 22 studies in adults from 13 different countries worldwide and reported weight gain in a pooled average of 32% of the population [1]. In Germany, the GEDA 2021 study reported a proportion of 26% [17], substantially lower than the 40% (S1) and 35% (S2) we found. A possible explanation for this discrepancy in findings is that the studies’ timings were different: the GEDA study was performed between July and October 2021, and thus, more time had passed since the restrictions of the winter lockdown compared to S1.

In the GEDA study, population subgroups with a BMI of ≥ 30 kg/m^2^ or age below 65 years were identified as most prone to weight gain. Our study's findings are similar with regard to BMI. With regard to age, the GEDA study demonstrated a continuous decrease in weight gain frequency with increasing age group, while in the present study, this decreasing trend was only seen starting in the age group of 30–44 years. The age group of 18–29 years demonstrated a lower weight gain frequency than the age group of 30–44 years.

Similar to the existing literature on the effects of the pandemic on body weight [24], we found weight loss to be less common than weight gain. Nevertheless, the average reported number of kilograms lost (6.4 kg in S1 and 7.9 kg in S2) was higher than the average number of kilograms gained (5.5 kg in S1 and 6.5 kg in S2). Of the entire sample, 46% (both surveys) of participants stated that they had tried to lose weight.

Our findings regarding dietary changes were heterogeneous and do not allow for solid conclusions on how people exactly changed their diets. However, the common finding of weight gain implies that a large proportion of people increased their caloric intake and/or decreased their physical activity level, resulting in a positive energy balance. A systematic review of 41 studies during the first year of the pandemic highlighted increased food intake and increased snacking as common findings [4]. The heterogeneity in healthier and unhealthier eating that we observed is supported by findings of another recent systematic review [1].

It is well-known that the COVID-19 pandemic gave rise to increased levels of stress, anxiety and depression in the general population [2]. Our observation that participants with moderate to severe mental stress more often reported unhealthier and emotional eating is in accordance with the literature on the relationship between stress or emotions and eating behavior [25–27]. Also during the COVID-19 pandemic, stress increased the risk of overeating and weight gain [5]. Whilst in our study, stress levels were lower in S2 than in S1, both surveys indicated that a majority of people felt moderately or severely stressed by the pandemic. Of note is that mental stress demonstrated a significant association with weight gain in the Chi-square tests, but not in the logistic regression model. This suggests that mental stress had an impact on weight gain through inducing changes in dietary behavior, since nutrition-related variables did show an association with weight gain.

This study comes with some limitations. The two surveys included independent samples, and thus, we could not test for longitudinal changes in the participants’ responses. Nevertheless, we were able to compare the two surveys in a valuable and meaningful way because both samples, with approximately 1,000 participants each, represented the general population of Germany. Another limitation is that all data were self-reported by the participants and most of the data were collected in a retrospective manner, introducing bias originating from the capacity to remember the situation before the pandemic. In addition, data on BMI was obtained by asking the participants about their current BMI, not pre-pandemic BMI. Ideally, the logistic regression model would have included pre-pandemic BMI. Also, bias introduced by the interest of the participants in the topic cannot be excluded. Finally, we did not take ethnicity into account [5, 28].

## Conclusions

In conclusion, the adverse effects of the COVID-19 pandemic on the lifestyle and body weight of adults in Germany were of a long-lasting sort. According to our analysis, more than a third of the population gained weight, which was mirrored by reported reductions in physical activity and changes in dietary behavior. Considering the increased health risks of overweight and obesity, these findings ask for targeted strategies to better support those population subgroups that are most prone to unfavorable lifestyle changes when faced with severe disruptions of their everyday lives. Further follow-up studies are needed to determine whether these adverse sequelae of the COVID-19 pandemic are reversible.

### Supplementary Information

**Supplementary Material 1.**

**Supplementary Material 2.**

## Data Availability

The data presented in this study are not publicly available. All data generated or analyzed during this study are included in this article. Further inquiries can be directed to the corresponding author. The original data are available from the corresponding author upon reasonable request.

## Abbreviations

BMI: Body mass index
S1: Survey 1
S2: Survey 2
AOR: Adjusted odds ratio
