# Supplementary material for: Long-lasting effects of the COVID-19 pandemic on lifestyle and body weight: results of representative cross-sectional surveys in adults in Germany
Source: BMC Public Health. 2024 Apr 30;24:1199. doi: 10.1186/s12889-024-18680-x (PMC11059715; doi:10.1186/s12889-024-18680-x)
Supplement: Supplementary file 1 — Supplementary Material 1. [file 12889_2024_18680_MOESM1_ESM.pdf]

Long-lasting effects of the COVID-19 pandemic on lifestyle and body weight: results of representative cross-sectional surveys in adults in Germany

Hans Hauner[1,2], Carmen P.S. Blanken[1], Christina Holzapfel[1,3]

[1]Institute of Nutritional Medicine, School of Medicine and Health, Technical University of Munich, Munich, Germany.
[2]Else Kröner Fresenius Center for Nutritional Medicine, Technical University of Munich, Munich, Germany.
[3]Department of Nutritional, Food and Consumer Sciences, Fulda University of Applied Sciences, Fulda, Germany.

Supplementary material

QUESTIONNAIRE "Lifestyle and nutrition in the COVID-19 pandemic"
1,000 people between the ages of 18 and 70 years

- Translated from German -

S1/S2: The following survey contains questions about everyday life and nutrition during the COVID-19 period. First, we ask you to answer a few general questions about your household and your family situation.

1. S1/S2: Do you live together with a partner or spouse in your household?

   - yes
   - no
   - no comment

2. S1/S2: How many children under the age of 18 years live in your household?

   - 1 child
   - 2 children
   - 3 or more children
   - no child
   - no comment

3. S1/S2: Has your job changed <u>since the start of the COVID-19 pandemic?</u>

   Single answer possible (always at the beginning)
   - I have not been employed since the beginning of the COVID-19 pandemic

   Multiple answers possible
   - I have lost my former job
   - I am or was employed with reduced working hours
   - I am or was mainly in home office
   - I am or was partly in home office
   - my employer changed
   - I increased my working hours
   - I reduced my working hours

   Single answer possible (always at the end)
   - there has not been a change
   - nothing above applies to me
   - no comment

4. S1: How strongly do you feel mentally stressed by the changes in relation to the COVID-19 situation?
   S2: How strongly did you, in the past year, feel mentally stressed by the changes in relation to the COVID-19 situation?

   - severely
   - moderately
   - mildly
   - not at all
   - don't know / no comment

5. S1: Please think about the theme physical activity. Would you say you, since the beginning of the COVID-19 pandemic, altogether moved more or less or was there no substantial change?
   S2: Please think about the theme physical activity. Would you say you, in the past year, altogether moved more or less or was there no substantial change compared to the situation before the COVID-19 pandemic?

   - more than before
   - less than before
   - no change
   - don't know / no comment

IF Q5: LESS THAN BEFORE

6. S1: Why do you think you have been moving less since the beginning of the COVID-19 pandemic?
   S2: Why do you think you have, in the past year, been moving less compared to before the COVID-19 pandemic?

   Multiple answers possible, order of the answer options was randomized
   - facilities (e.g. gyms, fitness studios) for individual or group sports were closed
   - there were no or only a few offers for individual or group sports
   - I have less time for exercise
   - I have less desire to exercise
   - I move less in everyday life
   - I move less during work

   At the end of the block
   - other reasons

   Single answer possible (always at the end)
   - don't know / no comment

7. S1: In what way has your dietary behavior changed since the beginning of the COVID-19 pandemic? Please mark which of the statements most applies to you.
   S2: In what way has your dietary behavior changed compared to before the COVID-19 pandemic? Please mark which of the statements most applies to you.

   Order of the questions was randomized

   7.1
   - I eat healthier
   - I eat less healthy
   - no substantial change
   - don't know / no comment

   7.2
   - I eat more regularly
   - I eat less regularly
   - no substantial change
   - don't know / no comment

7.3

- I eat more main meals
- I eat fewer main meals
- no substantial change
- don't know / no comment

7.4

- I eat more snacks
- I less fewer snacks
- no substantial change
- don't know / no comment

7.5

- I eat larger portions
- I eat smaller portions
- no substantial change
- don't know / no comment

7.6

- I have an increased appetite
- I have a decreased appetite
- no substantial change
- don't know / no comment

7.7

- I have less time to eat
- I have more time to eat
- no substantial change
- don't know / no comment

7.8

- I eat more often out of boredom
- I eat less often out of boredom
- no substantial change
- don't know / no comment

7.9

- I use food more often as a reward
- I use food less often as a reward
- no substantial change
- don't know / no comment

8. S1: You will see some photos of food items and meals. Please indicate whether you, since the beginning of the COVID-19 pandemic, have eaten more, less, or equal amounts of the following food groups.
S2: You will see some photos of food items and meals. Please indicate whether you, in the past year compared to before the COVID-19 pandemic, have eaten more, less, or equal amounts of the following food groups.

- more
- less
- equal amounts
- don't know / no comment

Order of the answer options was randomized

- Sweets (e.g. chocolate, chocolate bars, ice cream)
- Cakes and pastries (e.g. cookies, cakes, pastries)

- Crisps (e.g. potato chips, peanut puffs, pretzel sticks)
- Fast food (e.g. burgers, pizza, kebab)
- Fruit
- Vegetables
- Alcohol
- Coffee
- Soft drinks (e.g. cola, soft drinks, iced tea)

9. S1: Please indicate whether you, since the beginning of the COVID-19 pandemic, have done the following things more often, less often or just as often as before.
S2: Please indicate whether you, in the past year compared to before the COVID-19 pandemic, have done the following things more often, less often or just as often as before.

- more often
- less often
- just as often / no change
- don't know / no comment

Order of the answer options was randomized
- Cooking / preparing meals
- Ordering meal delivery
- Getting take-out meals
- Grocery shopping

10. S1/S2: Has your body weight changed since the beginning of the COVID-19 pandemic?

- yes, I have gained weight
- yes, I have lost weight
- no
- don't know / no comment

IF Q10: GAINED OR LOST
11. S1/S2: How many kilos have you since the beginning of the COVID-19 pandemic
IF Q10: GAINED
gained?
IF Q10: LOST
lost?
Please enter this in entire kilograms.

NUMBER FIELD KILOGRAMS (limits: 1 to 50)
- don't know / no comment

12. S1/S2: How tall are you? Please enter your height in cm.

HEIGHT IN CM (limits: 130 cm to 230 cm)
- don't know / no comment

13. S1/S2: And how much do you currently weigh? Please enter your weight in kg.

WEIGHT IN KG (limits: 30 to 200)
- don't know / no comment

14. S1/S2: Have you tried to reduce your body weight in the past six months?

- yes
- no
- no comment

IF Q14: YES

15. S1/S2: How have you tried to reduce your body weight? What applies to you?

Multiple answers possible, order of the answer options was randomized
- I went on a diet
- I have changed my diet
- I have engaged in more activity
- I have used medication/aids for weight loss
- I have used an app for weight loss

At the end of the block
- other

Single answer possible (always at the end)
- don't know / no comment

Statistical data (available via master data)

- Age
- Gender
- Secondary education level
- Net household income
- Region (East-/West-Germany)
- Town size
