# Supplementary material for: Long-lasting effects of the COVID-19 pandemic on lifestyle and body weight: results of representative cross-sectional surveys in adults in Germany
Source: BMC Public Health. 2024 Apr 30;24:1199. doi: 10.1186/s12889-024-18680-x (PMC11059715; doi:10.1186/s12889-024-18680-x)
Supplement: Supplementary file 2 — Supplementary Material 2. [file 12889_2024_18680_MOESM2_ESM.pdf]

Long-lasting effects of the COVID-19 pandemic on lifestyle and body weight: results of representative cross-sectional surveys in adults in Germany

Hans Hauner[1,2], Carmen P.S. Blanken[1], Christina Holzapfel[1,3]

[1]Institute of Nutritional Medicine, School of Medicine and Health, Technical University of Munich, Munich, Germany.
[2]Else Kröner Fresenius Center for Nutritional Medicine, Technical University of Munich, Munich, Germany.
[3]Department of Nutritional, Food and Consumer Sciences, Fulda University of Applied Sciences, Fulda, Germany.

Supplementary Tables

Supplementary Table 1. Proportions of survey participants reporting changes in body weight, mental stress levels, physical activity levels, healthier/unhealthier nutrition and consumption of energy-dense food groups, stratified by survey. Chi-square test results are included in the form of p-values, with p-values of <.05 (displayed in bold) indicating a significant association. 95%-confidence intervals are indicated in brackets.

	Body weight		
Survey	Gained weight	No change/lost weight	p
S1 (N=1,001)	40% [37%, 43%]	60% [57%, 63%]	0.059
S2 (N=1,005)	35% [32%, 39%]	65% [61%, 68%]	
	Mental stress		
	Not at all/mild	Moderate/severe	p
S1 (N=1,001)	29% [26%, 32%]	71% [68%, 74%]	**<.001**
S2 (N=1,005)	38% [35%, 41%]	62% [59%, 65%]	
	Physical activity		
	Reduced	No change/increased	p
S1 (N=1,001)	52% [49%, 56%]	48% [44%, 51%]	**<.001**
S2 (N=1,005)	40% [37%, 44%]	60% [56%, 63%]	
	Healthy nutrition		
	Less healthy	No change/more healthy	p
S1 (N=1,001)	16% [14%, 18%]	84% [82%, 86%]	**0.033**
S2 (N=1,005)	12% [10%, 15%]	88% [85%, 90%]	
	Energy-dense food consumption		
	More	No mean change/less	p
S1 (N=1,001)	32% [29%, 35%]	68% [65%, 71%]	0.060
S2 (N=1,005)	27% [24%, 31%]	73% [69%, 76%]	

Supplementary Table 2. Proportions of survey participants reporting no or mild mental stress versus moderate to severe mental stress, stratified by demographic and socioeconomic variables. Chi-square test results are included in the form of p-values, with p-values of <.05 (displayed in bold) indicating a significant association between the variable and mental stress status. 95%-confidence intervals are indicated in brackets.

		Survey S1 (N = 1,001)			Survey S2 (N = 1,005)		
		No/mild mental stress (n=292)	Moderate/severe mental stress (n=707)	p	No/mild mental stress (n=379)	Moderate/severe mental stress (n=626)	p
Gender	Men	35% [31%, 40%]	65% [60%, 69%]	**<.001**	45% [41%, 50%]	55% [50%, 59%]	**<.001**
	Women	23% [19%, 27%]	77% [73%, 81%]		30% [26%, 35%]	70% [65%, 74%]	
Age (years)	18-29	24% [17%, 33%]	76% [67%, 83%]	0.396	29% [21%, 39%]	71% [61%, 79%]	**<.001**[a]
	30-44	29% [23%, 35%]	71% [65%, 77%]		32% [26%, 38%]	68% [62%, 74%]	
	45-59	31% [26%, 35%]	69% [65%, 74%]		40% [36%, 45%]	60% [55%, 64%]	
	60-70	33% [26%, 40%]	67% [60%, 74%]		51% [43%, 58%]	49% [42%, 57%]	
Body mass index (kg/m^2)	<25	31% [26%, 37%]	69% [63%, 74%]	0.279	39% [33%, 44%]	61% [56%, 67%]	**0.004**[b]
	25 to <30	26% [21%, 32%]	74% [68%, 79%]		43% [37%, 49%]	57% [51%, 63%]	
	≥30	32% [26%, 39%]	68% [61%, 74%]		29% [23%, 35%]	71% [65%, 77%]	
Secondary education level	General	32% [26%, 39%]	68% [61%, 74%]	0.458	43% [36%, 49%]	57% [51%, 64%]	**<.001**[c]
	Intermediate	29% [24%, 35%]	71% [65%, 76%]		47% [41%, 53%]	53% [47%, 59%]	
	High	27% [23%, 32%]	73% [68%, 77%]		30% [25%, 35%]	70% [65%, 75%]	
Net household income (euros)	<2,000	31% [24%, 38%]	69% [62%, 76%]	0.562	38% [30%, 46%]	62% [54%, 70%]	0.719
	2,000 to <4,000	31% [26%, 37%]	69% [63%, 74%]		39% [35%, 45%]	61% [55%, 65%]	
	≥4,000	27% [22%, 33%]	73% [67%, 78%]		36% [31%, 42%]	64% [58%, 69%]	

a) Significant difference between age groups 18-29 years and 45-59 years ($p=0.039$), 18-29 years and 60-70 years ($p<.001$), and 30-44 years and 60-70 years ($p<.001$).
b) Significant difference between body mass index (BMI) groups <25 kg/m^2 and ≥30 kg/m^2 ($p=0.021$), and 25 to <30 kg/m^2 and ≥30 kg/m^2 ($p=0.001$).
c) Significant difference between secondary education levels general and high ($p=0.002$), and intermediate and high ($p<.001$).

Supplementary Table 3. Proportions of survey participants reporting reduced physical activity versus no change/increased physical activity, stratified by demographic and socioeconomic variables. Chi-square test results are included in the form of p-values, with p-values of <.05 (displayed in bold) indicating a significant association between the variable and physical activity status. 95%-confidence intervals are indicated in brackets.

		Survey S1 (N = 1,001)			Survey S2 (N = 1,005)		
		Reduced physical activity (n=521)	No change/increased physical activity (n=477)	p	Reduced physical activity (n=404)	No change/increased physical activity (n=598)	p
Gender	Men	55% [51%, 60%]	45% [40%, 49%]	0.058	36% [31%, 40%]	64% [60%, 69%]	**0.007**
	Women	49% [44%, 54%]	51% [46%, 56%]		45% [40%, 50%]	55% [50%, 60%]	
Age (years)	18-29	56% [47%, 65%]	44% [35%, 53%]	0.249	47% [38%, 57%]	53% [43%, 62%]	**<.001**[c]
	30-44	56% [49%, 62%]	44% [38%, 51%]		48% [41%, 54%]	52% [46%, 59%]	
	45-59	49% [44%, 54%]	51% [46%, 56%]		38% [33%, 43%]	62% [57%, 67%]	
	60-70	48% [41%, 56%]	52% [44%, 59%]		27% [21%, 33%]	73% [67%, 79%]	
Body mass index (kg/m^2)	<25	49% [43%, 54%]	51% [46%, 57%]	**0.050**[a]	35% [30%, 41%]	65% [59%, 70%]	**0.009**[d]
	25 to <30	54% [48%, 60%]	46% [40%, 52%]		39% [33%, 45%]	61% [55%, 67%]	
	≥30	60% [53%, 66%]	40% [34%, 47%]		49% [42%, 56%]	51% [44%, 58%]	
Secondary education level	General	47% [40%, 54%]	53% [46%, 60%]	**0.005**[b]	35% [29%, 42%]	65% [58%, 71%]	**<.001**[e]
	Intermediate	50% [44%, 56%]	50% [44%, 56%]		33% [28%, 39%]	67% [61%, 72%]	
	High	60% [55%, 65%]	40% [35%, 45%]		48% [43%, 54%]	52% [46%, 57%]	
Net household income (euros)	<2,000	57% [49%, 65%]	43% [35%, 51%]	0.416	33% [26%, 42%]	67% [58%, 74%]	0.104
	2,000 to <4,000	51% [45%, 56%]	49% [44%, 55%]		41% [36%, 46%]	59% [54%, 64%]	
	≥4,000	52% [46%, 58%]	48% [42%, 54%]		44% [38%, 51%]	56% [49%, 62%]	

a) Significant difference between Body mass index (BMI) groups <25 kg/m^2 and ≥30 kg/m^2 (p=0.022).

b) Significant difference between secondary education levels general and high ($p=0.003$), and intermediate and high ($p=0.011$).
c) Significant difference between age groups 18-29 years and 60-70 years ($p<.001$), 30-44 years and 60-70 years ($p<.001$), and 45-59 years and 60-70 years ($p=0.038$).
d) Significant difference between body mass index (BMI) groups <25 kg/m^2 and ≥30 kg/m^2 ($p=0.002$), and 25 to <30 kg/m^2 and ≥30 kg/m^2 ($p=0.036$).
e) Significant difference between secondary education levels general and high ($p=0.002$), and intermediate and high ($p<.001$).

Supplementary Table 4. Proportions of survey participants reporting less healthy nutrition versus no change/more healthy nutrition, stratified by demographic and socioeconomic variables. Chi-square test results are included in the form of p-values, with p-values of <.05 (displayed in bold) indicating a significant association between the variable and healthy nutrition status. 95%-confidence intervals are indicated in brackets.

		Survey S1 (N = 1,001)			Survey S2 (N = 1,005)		
		Less healthy nutrition (n=159)	No change/more healthy nutrition (n=839)	p	Less healthy nutrition (n=123)	No change/more healthy nutrition (n=881)	p
Gender	Men	15% [12%, 19%]	85% [81%, 81%]	0.706	14% [11%, 17%]	86% [83%, 89%]	0.236
	Women	16% [13%, 20%]	84% [80%, 87%]		11% [8.0%, 14%]	89% [86%, 92%]	
Age (years)	18-29	18% [12%, 26%]	82% [74%, 88%]	**0.002**[a]	15% [9.4%, 24%]	85% [76%, 91%]	**0.004**[c]
	30-44	21% [16%, 27%]	79% [73%, 84%]		17% [13%, 23%]	83% [77%, 87%]	
	45-59	15% [12%, 19%]	85% [81%, 88%]		10% [7.2%, 13%]	90% [87%, 93%]	
	60-70	7.1% [4.2%, 12%]	93% [88%, 96%]		5.9% [3.3%, 10%]	94% [90%, 97%]	
Body mass index (kg/m^2)	<25	13% [10%, 17%]	87% [83%, 90%]	**0.014**[b]	6.5% [4.2%, 10%]	94% [90%, 96%]	**0.001**[d]
	25 to <30	14% [11%, 19%]	86% [81%, 89%]		14% [10%, 19%]	86% [81%, 90%]	
	≥30	22% [17%, 28%]	78% [72%, 83%]		17% [13%, 23%]	83% [77%, 87%]	
Secondary education level	General	13% [9%, 18%]	87% [82%, 91%]	0.122	10% [6.2%, 16%]	90% [84%, 94%]	0.100
	Intermediate	14% [11%, 19%]	86% [81%, 89%]		10% [6.6%, 14%]	90% [86%, 93%]	
	High	19% [15%, 24%]	81% [76%, 85%]		15% [11%, 20%]	85% [80%, 89%]	
Net household income (euros)	<2,000	14% [9.3%, 21%]	86% [79%, 91%]	0.692	15% [10%, 23%]	85% [77%, 90%]	**0.026**[e]
	2,000 to <4,000	17% [14%, 22%]	83% [78%, 86%]		9.0% [6.3%, 13%]	91% [87%, 94%]	
	≥4,000	16% [12%, 21%]	84% [79%, 88%]		17% [12%, 21%]	83% [79%, 88%]	

a) Significant difference between age groups 18-29 years and 60-70 years (p=0.007), 30-44 years and 60-70 years (p<.001), and 45-59 years and 60-70 years (p=0.028).
b) Significant difference between body mass index (BMI) groups <25 kg/m^2 and ≥30 kg/m^2 (p=0.009), and 25 to <30 kg/m^2 and ≥30 kg/m^2 (p=0.030).
c) Significant difference between age groups 18-29 years and 60-70 years (p=0.020), 30-44 years and 45-59 years (p=0.036), and 30-44 years and 60-70 years (p=0.002).
d) Significant difference between body mass index (BMI) groups <25 kg/m^2 and 25 to <30 kg/m^2 (p=0.002), and <25 kg/m^2 and ≥30 kg/m^2 (p<.001).
e) Significant difference between net household income of 2,000 to <4,000 euros and ≥4,000 euros (p=0.006).

Supplementary Table 5. Proportions of survey participants reporting increased consumption of energy-dense food groups versus no mean change/reduced consumption of energy-dense food, stratified by demographic and socioeconomic variables. Chi-square test results are included in the form of p-values, with p-values of <.05 (displayed in bold) indicating a significant association between the variable and energy-dense food group consumption. 95%-confidence intervals are indicated in brackets.

		Survey S1 (N = 1,001)			Survey S2 (N = 1,005)		
		Increased energy-dense food group consumption (n=316)	No mean change/ reduced consumption (n=685)	p	Increased energy-dense food group consumption (n=274)	No mean change/ reduced consumption (n=731)	p
Gender	Men	30% [26%, 34%]	70% [66%, 74%]	0.281	26% [22%, 31%]	74% [69%, 78%]	0.549
	Women	33% [29%, 38%]	67% [62%, 71%]		28% [24%, 33%]	72% [67%, 76%]	
Age (years)	18-29	33% [25%, 42%]	67% [58%, 75%]	**<.001**[a]	35% [26%, 45%]	65% [55%, 74%]	**<.001**[d]
	30-44	41% [35%, 47%]	59% [53%, 65%]		37% [30%, 43%]	63% [57%, 70%]	
	45-59	32% [27%, 37%]	68% [63%, 73%]		23% [19%, 27%]	77% [73%, 81%]	
	60-70	16% [12%, 22%]	84% [78%, 88%]		14% [10%, 20%]	86% [80%, 90%]	
Body mass index (kg/m^2)	<25	27% [22%, 32%]	73% [68%, 78%]	**0.001**[b]	24% [20%, 30%]	76% [70%, 80%]	0.075
	25 to <30	29% [24%, 35%]	71% [65%, 76%]		27% [21%, 32%]	73% [68%, 79%]	
	≥30	42% [36%, 49%]	58% [51%, 64%]		34% [28%, 41%]	66% [59%, 72%]	
Secondary education level	General	26% [21%, 32%]	74% [68%, 79%]	**0.033**[c]	22% [17%, 29%]	78% [71%, 83%]	**0.001**[e]
	Intermediate	31% [26%, 37%]	69% [63%, 74%]		22% [17%, 28%]	78% [72%, 83%]	
	High	37% [32%, 42%]	63% [58%, 68%]		35% [30%, 41%]	65% [59%, 70%]	
Net household income (euros)	<2,000	31% [24%, 39%]	69% [61%, 76%]	0.150	20% [14%, 28%]	80% [72%, 86%]	**<.001**[f]
	2,000 to <4,000	29% [25%, 34%]	71% [66%, 75%]		23% [19%, 28%]	77% [72%, 81%]	
	≥4,000	37% [31%, 43%]	63% [57%, 69%]		37% [31%, 43%]	63% [57%, 69%]	

a) Significant difference between age groups 18-29 years and 60-70 years ($p<.001$), 30-44 years and 60-70 years ($p<.001$), and 45-59 years and 60-70 years ($p<.001$).
b) Significant difference between body mass index (BMI) groups <25 kg/m^2 and ≥30 kg/m^2 ($p<.001$), and 25 to <30 kg/m^2 and ≥30 kg/m^2 ($p=0.002$).
c) Significant difference between secondary education levels general and high ($p=0.016$).
d) Significant difference between age groups 18-29 years and 45-59 years ($p=0.010$), 18-29 years and 60-70 years ($p<.001$), 30-44 years and 45-59 years ($p=0.001$), 30-44 years and 60-70 years ($p<.001$), and 45-59 years and 60-70 years ($p=0.047$).
e) Significant difference between secondary education levels general and high ($p=0.002$), and intermediate and high ($p<.001$).
f) Significant difference between net household income of <2,000 euros and ≥4,000 euros ($p<.001$), and 2,000 to <4,000 euros and ≥4,000 euros ($p<.001$).

Supplementary Table 6. Proportions of survey participants reporting changes in body weight, physical activity levels, healthier/unhealthier nutrition and consumption of energy-dense food groups, stratified by mental stress level. Chi-square test results are included in the form of p-values, with p-values of <.05 (displayed in bold) indicating a significant association. 95%-confidence intervals are indicated in brackets.

	Survey S1 (N = 1,001)			Survey S2 (N = 1,005)		
	Body weight					
	Gained weight (n=389)	No change/lost weight (n=588)	p	Gained weight (n=352)	No change/lost weight (n=645)	p
No/mild mental stress	32% [27%, 38%]	68% [62%, 73%]	**0.004**	23% [19%, 28%]	77% [72%, 81%]	**<0.001**
Moderate/severe mental stress	43% [39%, 47%]	57% [53%, 61%]		42% [38%, 47%]	58% [53%, 62%]	
	Physical activity					
	Reduced (n=521)	No change/increased (n=477)	p	Reduced (n=404)	No change/increased (n=598)	p
No/mild mental stress	43% [37%, 49%]	57% [51%, 63%]	**0.001**	25% [20%, 30%]	75% [70%, 80%]	**<0.001**
Moderate/severe mental stress	56% [52%, 60%]	44% [40%, 48%]		50% [45%, 54%]	50% [46%, 55%]	
	Healthy nutrition					
	Less healthy (n=159)	No change/more healthy (n=839)	p	Less healthy (n=123)	No change/more healthy (n=881)	p
No/mild mental stress	6.1% [3.7%, 10%]	94% [90%, 96%]	**<0.001**	6.6% [4.2%, 10%]	93% [90%, 96%]	**<0.001**
Moderate/severe mental stress	20% [17%, 23%]	80% [77%, 83%]		16% [13%, 19%]	84% [81%, 87%]	
	Energy-dense food consumption					
	More (n=316)	Less/no mean change (n=685)	p	More (n=274)	Less/no mean change (n=731)	p
No/mild mental stress	20% [15%, 25%]	80% [75%, 85%]	**<0.001**	16% [12%, 21%]	84% [79%, 88%]	**<0.001**
Moderate/severe mental stress	36% [33%, 40%]	64% [60%, 67%]		34% [30%, 39%]	66% [61%, 70%]	
